# Can Inflammatory Markers Foretell Aetiology and Prolonged Hospitalisation in Acute Pancreatitis?

**DOI:** 10.7759/cureus.12566

**Published:** 2021-01-07

**Authors:** Umasankar Mathuram Thiyagarajan, Amirthavarshini Ponnuswamy, Rhys Thomas

**Affiliations:** 1 Department of Hepatobiliary and Pancreatic Surgery, Addenbrooke's Hospital, Cambridge University Hospitals NHS Foundation Trust, Cambridge, GBR; 2 Department of Family Medicine, Kent, Surrey and Sussex Deanery, Epsom, GBR; 3 Department of General Surgery, Croydon University Hospital, Thornton Heath, GBR

**Keywords:** pancreatitis, acute abdominal pain, gallstones, c-reactive protein, morbidity, mortality

## Abstract

Introduction

Acute pancreatitis (AP) causes a cascade of complex inflammatory responses following an initial insult. Hence, the scoring systems include white blood cell count (WBC) as a marker of severity of acute pancreatitis. C-reactive protein (CRP) was also shown to be useful in predicting the course of pancreatitis. This study analyses role of inflammatory markers in predicting gallstone aetiology of AP and length of hospital stay (LOS).

Materials and methods

A total of 143 patients with acute pancreatitis between October 2016 and 2017 were included in this study and relevant parameters were collected from the electronic patient database. The parameters were WBC, CRP, and LOS.

Results

Among 143 patients with AP, 50 patients had gallstone pancreatitis (GP) and remaining of 93 patients suffered nongallstone pancreatitis (NGP). The WBC count at admission, 24 hours and 72 hours in GP versus NGP were 11.6± 5 versus 13.7±17; P = 0.24; 12.6±20 versus 10.1±17; P = 0.21; and 13.2±22 versus 9.2±4.7; P = 0.15, respectively. Similarly, the serum CRP levels at admission, 24 hours and 72 hours were 30.4± 73 versus 47.6±79; P = 0.25; 71.9±20 versus 92.2±97; P = 0.35; and 89±106 versus 122.7±107; P = 0.05, respectively. More number of patients with elevated WBC in GP arm compared to NGP (12/50±7/93; P = 0.0008) was noted.

In GP arm, patients with elevated CRP at admission (10.5±8.67 versus 5.4±5.8 days; P = 0.02) and 24 hours (9.8±8.3 versus 4.2±4.7 days; P = 0.001) had long LOS. However, patients with elevated CRP at 72 hours (89±106 versus 122.7±107; P = 0.05) had longer LOS in NGP.

Conclusion

Significantly high CRP level at 72 hours was associated with NGP and longer length of hospital stay. In GP, patients with elevated CRP level at admission and 24 hours predicts long LOS.

## Introduction

Acute pancreatitis (AP) is a well-known and a common cause of emergency hospital admission. Worldwide, the incidence of AP varies between 4.9 and 73.4 cases per 100,000 [1,2]. In the UK, the approximate incidence ranges from 15 to 42 cases per 100,000 population [3,4]. In the United States, AP was the most common gastroenterology discharge diagnosis with a cost of 2.6 billion dollars in 2009 [1].To date, the incidence of AP in most of the developing countries including southeast Asia is not available in the literature.

Pathophysiology of AP

In western countries, gallstone diseases and alcohol abuse are the two most common causes of AP [5,6]. Slippage of small gallstones to the common bile duct causes a blockage, usually at the ampulla of Vater; this leads to obstruction of pancreatic duct thereby activation of enzymes. Alcohol is a direct toxin to pancreatic acinar cells and also causes premature activation of digestive enzymes leading to autodigestive injury [7]. Both conditions cause a cascade of inflammatory response after activation of pancreatic enzymes in the pancreas, called AP [8,9].

Other rare causes of AP include instrumentation of biliopancreatic duct such as endoscopic retrograde cholangiopancreatography (ERCP), trauma, hypercalcemia, drug-induced and certain venoms including scorpion; the genetic causes like cystic fibrosis, PRSS1, SPINK1 mutations and CTRB1-CTRB2 locus inversion have been also reported [10]. The success of management of AP depends on identifying the aetiology early to treat and prevent recurrences.

Although the majority will have a mild form of pancreatitis with a good prognosis, there are still 20%-30% of patients who develop a serious form of the disease with infection, sepsis and organ failure [11]. Among the patients with severe AP, 10%-20% will die [11]. Hence, it becomes essential to identify those who have the potential to develop a severe form of AP at the early stage, more specifically the inflammatory markers.

Inflammatory markers in AP

C-reactive protein (CRP) is an acute-phase reactant shown to be raised in inflammatory, infective, neoplastic conditions and after significant tissue injury [12]. CRP is synthesised in the liver after induction of interleukin-1 (IL-1) and interleukin-6 (IL-6) and it has been used to assess the severity of inflammatory response [13-17], The use of CRP in pancreatitis as a marker to assess the course of the disease had been there since the 1980s [15,18]. However, in a complex inflammatory response like AP, how the CRP response can be utilised to predict the aetiology is yet to be established.

The white blood cell count (WBC) at admission was shown to be elevated in AP and has a predictive value on severe AP [19]. WBC count in pancreatitis was never investigated as a marker to predict the aetiology, course of the disease, and length of hospital stay.

## Materials and methods

Patients who have been diagnosed with AP at our tertiary care hospital during the one-year period from October 2016 to 2017 were included in this study. The study conformed to the ethical guidelines of the 1975 Declaration of Helsinki. Due to the nature of the study, neither the ethical committee approval nor patients consent was advised in line with the hospital policy.

The diagnosis AP was established by the presence of 2 of 3 (Atlanta) criteria [20] below:

1. Upper abdominal pain, more specifically epigastric pain.

2. Serum lipase elevation ≥3 times the upper reference limit of normal (URL). 

3. Characteristic findings of pancreatitis on imaging - contrast-enhanced computed tomography (CECT scan), magnetic resonance imaging (MRI), or abdominal ultrasonography [20].

Among the 143 patients with AP, 50 patients were diagnosed with gallstone pancreatitis (GP) and the remaining 93 patients suffered nongallstone pancreatitis (NGP). GP was defined as imaging confirmed gallstones with the absence of any other possible aetiology for AP. Remaining AP patients without the presence of gallstones were categorised as NGP. All relevant parameters including demography, laboratory tests, radiological investigations and length of hospital stay (LOS) were retrieved from a prospective recorded electronic patient database. 

All of our patients had serum lipase (reference range: 0-50 U/l), full blood count, bone profile, liver function tests including ALT, ALP, bilirubin, urea and electrolytes and lactate dehydrogenase when attending the hospital every day till being discharged.

All patients with AP underwent abdominal ultrasonography to rule out gallstone disease; patients with deranged liver function, dilated bile duct, or high suspicion of bile duct stones had magnetic resonance cholangiopancreatography (MRCP) in line with our local hospital guidelines. CECT scan of the abdomen was performed in all patients with severe AP (by using Ranson or Modified Imrie score of 3 or more) and when there was a diagnostic uncertainty (Table 1) [21]. Endoscopic retrograde cholangiopancreatography (ERCP) was performed within the same hospital admission in patients with jaundice, bile duct stones and cholangitis.

**Table 1:** Demographic parameters. ^Ѱ^Values in mean ± standard deviation. WBC: white blood cell count; CRP: C-reactive protein; LOS: length of stay at hospital; MRCP: magnetic resonance cholangiopancreatography; CT: computerised tomography; ASA: American Society of Anaesthesiologist physical status classification; AP: acute pancreatitis.

	Gallstone pancreatitis (n = 50)	Nongallstone pancreatitis (n = 93)	P-value
Age (years)^Ѱ^	52±19	51.9±15.9	0.90
Gender (M:F)	21:29	62:31	0.07
ASA 3 or more	28/50	62/93	0.27
WBC at admission^Ѱ^	11.89±3.5	11.3±5.01	0.24
WBC at 24 hrs^Ѱ^	12.6 ±2	10.1 ±4.7	0.21
WBC at 72 hrs^Ѱ^	13.2±2.2	9.2±4.7	0.15
Patients with leucocytosis at 72hrs	12/50	7/93	0.0008
CRP at admission^Ѱ^	30.4±73	47.6±79	0.25
CRP at admission ASA >3	28.3±33	74.7±82	0.02
CRP at 24 hrs^Ѱ^	71.9±20	92.2±97	0.35
CRP at 72 hrs^Ѱ^	89±106	122.7±107	0.05
CRP at 72 hours ASA >3	79.3±96	169±115	0.02
MRCP scan	33/50	34/93	0.85
CT scans	17/50	59/93	0.85
Severe AP Imrie/Ranson Score >3	19/50	44/93	0.25
ERCP	20/50	5/93	0.0001
LOS^Ѱ^	6.2±6	3.4±9.3	0.001
30-day mortality	0/50	2/93	0.54
MRCP scan	33/50	34/93	0.85

Exclusion criteria

The exclusion criterion was patients with a previous history of gallstone pancreatitis or bile duct stones/biliary disease, age below 18 years, and pregnancy. 

Statistical analysis

Data were recorded in an Excel spreadsheet (Microsoft, Redmond, WA) and GraphPad Instat version 3.06 (GraphPad Software, Inc, San Diego, CA). Descriptive statistics for continuous variables were recorded as mean ± SD or median (range) according to whether or not they were normally distributed. Normality testing was performed using the Kolmogorov-Smirnov test. Comparison between groups was performed with the Student’s t-test for parametric variables and the Mann-Whitney test for non-parametric variables. Categorical variables were analysed using the Chi-squared test with Yates correction or Fisher’s exact test. Statistical significance was defined as P < 0.05.

## Results

Among the 143 patients with AP, 50 of them (50/143- 35%; Table 1) have been diagnosed with GP and the remaining (93/143- 65%; Table1) were of NGP. Interestingly the majority of patients in our study were of NGP aetiology. Within the GP group, 21 were males and 29 were females; similarly 62 males, 31 females in NGP group (P = 0.07).

Demographic parameters between GP and NGP are comparable (Table1). Similar results noted at mean WBC, ASA 3 grade, MRCP and CT scan between GP and NGP (Table 1). As expected more patients in the GP arm had ERCP (20/50 versus 5/93; P=0.0001; Table 1) for bile duct obstruction. Incidence of severe AP between the GP and NGP (19/50 versus 44/93; P = 0.25; Table 1) Patients in GP arm had longer hospitalisation when compared to NGP (6.6±6 versus 3.4±9.3; P = 0.001; Table 1).

Varying response of CRP and WBC in AP

**Figure 1:**
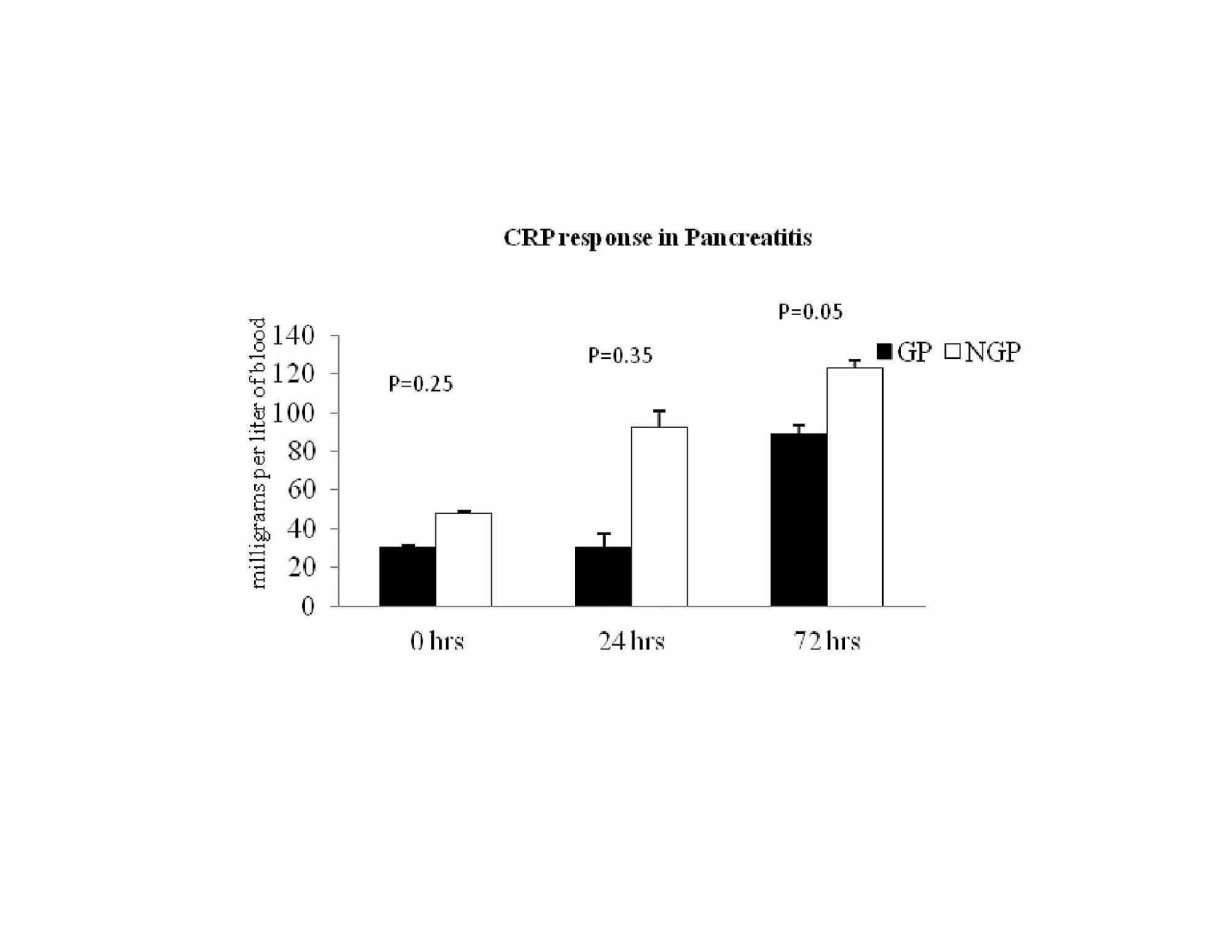
Graph 1: CRP response between GP versus NGP. CRP at 72 hours was significantly high in NGP (P-value <0.05). CRP: C-reactive protein; GP: gallstone pancreatitis; NGP: nongallstone pancreatitis.

We have analysed CRP level, WBC count to assess the role of these markers in predicting the aetiology of AP. Patients with normal CRP versus elevated CRP levels have been compared at the time of admission, 24 hour, and 48 hours. The mean CRP level at admission (30.4±73 versus 47.6±79; P = 0.25) and 24 hours (71.9±20 versus 92.2±97; P = 0.35) in GP versus NGP was not different (Figure 1 - Graph 1). However, patients with elevated CRP at 72 hours was associated with NGP (89±106 versus 122.7±107; P = 0.05; Figure 1 - Graph 1).

More interestingly, the patients with ASA3 exhibited a significantly high level of CRP at admission (28±33.4 versus 74.7±82; P = 0.02; Table 1) and 72 hours (79.3±96 versus 169±115; P = 0.02; Table 1). Similar finding was also noted in NGP where patients with ASA3 or more have shown high CRP levels compare to patients with ASA less than 3 (162±115 versus 73.7±85; P = 0.001). However, patients with ASA3 or more showed no difference compared to patients with less ASA grading (64±23 versus 79.3±96; P = 0.69) in GP.

**Figure 2:**
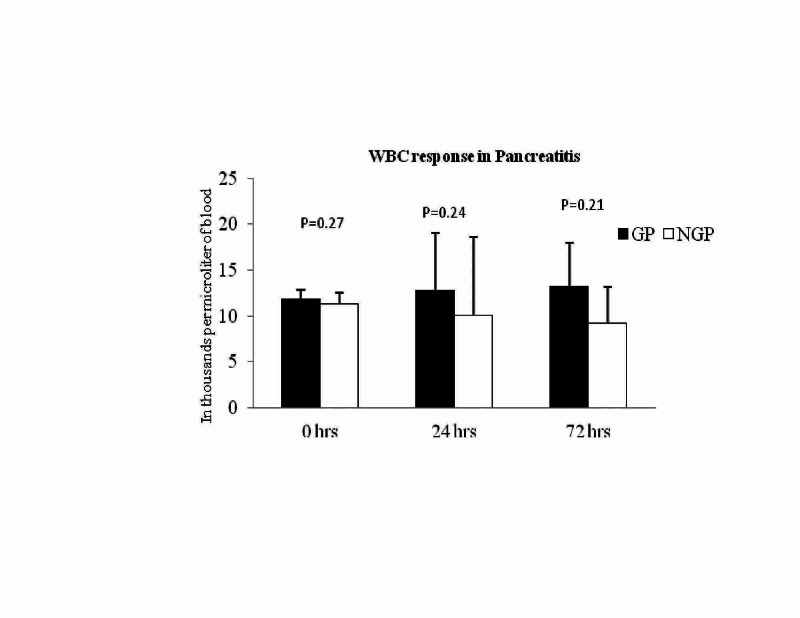
Graph 2: WBC response in acute pancreatitis. WBC response was not different between GP versus NGP from admission to 72 hours. GP: gallstone pancreatitis; NGP: nongallstone pancreatitis; WBC: white blood cell count.

The mean WBC level in AP was not different at admission (11.89±3.5 versus11.3±5.01; P = 0.24), 24 hours (12.6±2 versus 10.1 ±4.7; P = 0.21), and 72 hours (13.2±2.2 versus9.2±4.7; P = 0.15; Figure 2 - Graph 2) were not different between GP and NGP. Further analysis showed that no significant difference at the level of WBC between mild versus severe pancreatitis at 72 hours (8.09±1.4 versus 11±4; P = 0.44) However, significantly high number of patients with elevated WBC in the GP arm compared to NGP (12/50 versus 7/93; P = 0.0008; Table 1).

From NGP group of 93, the majority of them related to alcohol abuse (n = 87; 93.5%) and only 6 (6.5%) of them suffered from other causes including drug-induced trauma and idiopathic aetiology. Further analysis showed that no significant difference at the level of WBC between mild versus severe pancreatitis at 72 hours (9.07±3.4 versus 8.78±5.9; P = 0.83).

Predictivity of inflammatory markers on LOS

**Figure 3:**
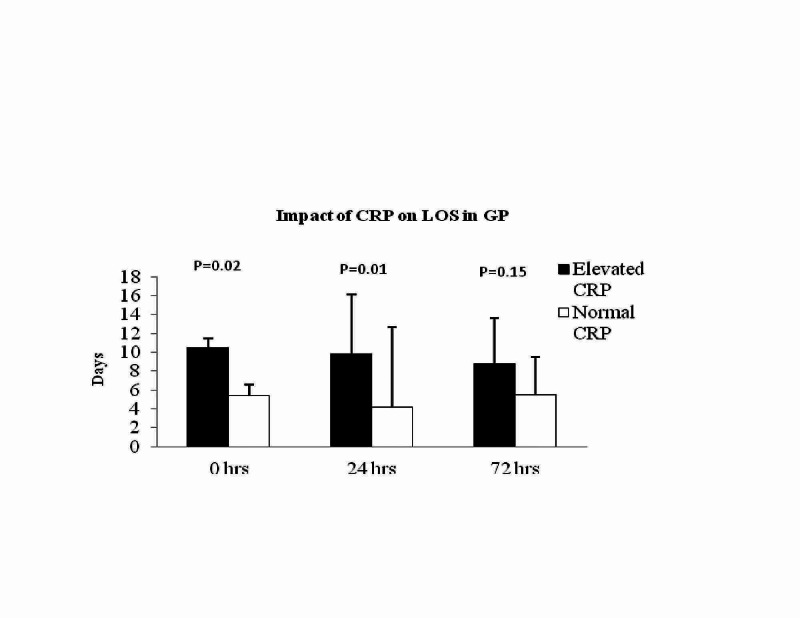
Graph 3: Impact of CRP on length of hospital stay (LOS) in GP. In GP, patients with high CRP levels at admission and 24 hours were associated with longer hospitalisation. CRP: C-reactive protein; GP: gallstone pancreatitis.

Among the GP cohort, patients with elevated CRP level at admission (10.5±8.67 versus 5.4±5.8 days; P = 0.02) and 24 hours (9.8±8.3 versus 4.2±4.7 days; P = 0.001; Figure 3 - Graph 3) had longer hospitalisation compared to the patients with normal levels. A similar result was observed in patients with elevated WBC count at 24 hours, staying more days at hospital (9.66±7.9 versus 5.2±6; P = 0.02). However, patients with elevated WBC at admission (7.26±7.3 versus 5.16 ±5.8 days; P = 0.13) and 72 hours (8.6±5.6 versus 7.6±8.2 days; P = 0.68) did not show any associated longer hospital stay.

**Figure 4:**
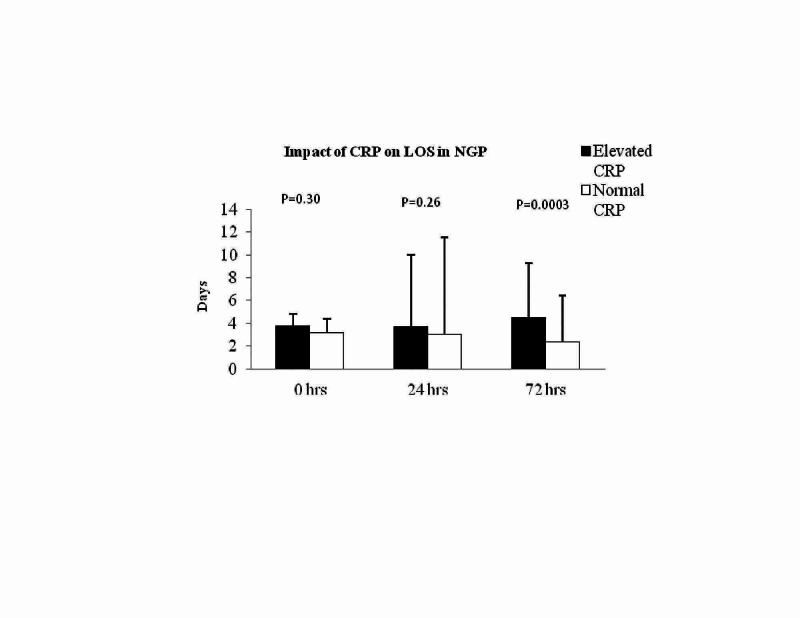
Graph 4: Impact of CRP on length of hospital stay in NGP. In NGP, patients with elevated CRP levels at 72 hours had longer hospital stay (P-value =0.0003). CRP: C-reactive protein; NGP: nongallstone pancreatitis.

In NGP arm, patients elevated CRP at admission (3.8±2.2 versus 3.6±2.2 days; P = 0.30; Figure 4 - Graph 4) and 24 hours (3.68±2 versus 3.03±2.6; P = 0.26; Figure 4 - Graph 4) was not associated with a longer hospital stay. However, patients with elevated CRP at 72 hours were found to have longer hospital stay (4.5±2.2 versus 2.48±1.9; P = 0.0003; Figure 4 - Graph 4). However, patients with elevated WBC did not have any impact on the length of hospital stay in NGP patients (Table 1). Mortality at 30 days between the GP versus NGP was not different (0/50 versus 2/93; P = 0.54; Table1).

 

## Discussion

Making the diagnosis of abdominal pain can be daunting when a surgeon wants to exclude conditions that require immediate intervention. There could be a place for confusion when there is a delay between onset of symptoms and presentation at the hospital [22]. Once the diagnosis of AP has been made, it becomes crucial to establish the aetiology as this would guide the next steps of definitive treatment. Although the immediate management of AP is not different, GP would require future planning for definitive management.

The UK guideline recommends all patients with GP should undergo definitive management of gallstones during the same hospital admission unless a plan is in place for definitive treatment [23]. It also recommends an urgent therapeutic ERCP to be performed in patients with suspected or proven gallstone aetiology where a predicted or actual severe pancreatitis, or when there is cholangitis, jaundice, or a dilated common bile duct [23]. Hence for the above reasons, establishing the aetiology is crucial in GP. In our study, more patients in GP arm had ERCP for bile duct stone; five out of 93 patients in NGP also needed ERCP for cholangitis/biliary obstruction secondary to bile duct stones (Table 1).

Establishing the role of inflammatory markers in predicting gallstone aetiology would be of immense value in the management of pancreatitis, in particular to developing countries where the resources are scanty. Previous studies have shown that amylase [24], serum ALP, total bilirubin, and lipase levels were significantly higher in GP [25].

There were some evidence to support that those patients with elevated CRP at 48 hours and at the end of a week are associated with severe AP and poor outcomes [11,15,26]. Hence, it is widely accepted that elevated level of CRP as a surrogate marker of severe pancreatitis and higher mortality [11]. To date, there is no evidence in the literature using high CRP in AP to predict the aetiology. Our study showed that patients with elevated CRP at 72 hours was associated with NGP (Table 1). More interestingly, the NGP patients with ASA3 also exhibited a significantly high level of CRP at admission and 72 hours; however, a similar finding was not seen in patients with ASA3 in GP cohort.

LOS is a crucial factor which has implication on the individual patient experience and the healthcare expenditure. LOS not only affects the day-to-day medical services but longer LOS was associated with side effects of medications and hospital-acquired infections [27,28]. Further to this, shorter hospital stay reduces the pressure on the healthcare workforce, improves the bed turnover and sustainability of public-funded hospitals and lowers the social costs too [29]. Having limited information to predict the hospital stay can affect the patient and doctor partnership which is an important factor in the care management with consequences on the quality of patient care.

There are a few limitations to our study. Firstly, the sample size in our study was small and it was a retrospective study. Secondly, though we performed the blood investigations at admission, 24 hours till discharge; we have included levels of inflammatory markers up to 72 hours due to the database limitations. Thirdly, the impact of antibiotics use was not considered in the study. Despite these limitations, there were strengths in our study too. We have retrieved the numbers of patients with severe pancreatitis, numbers of ERCPs, reviewed imaging, length of stay in hospital and mortality.

## Conclusions

In acute severe pancreatitis, there have been studies showing a significant reduction in physical and social functioning, general health and perceived wellbeing. Hence, it becomes essential to identify those patients who would require longer hospitalisation and providing the necessary information from the beginning. Our study showed that those patients with elevated CRP at admission and 24 hours have longer hospital stay in GP. However in NGP cohort, patients with elevated CRP at 72 hours were associated with longer hospital stay but this effect was not seen in patients with elevated CRP at admission and 24 hours (Figures 3, 4 - Graphs 3, 4). We believe that the above results can be helpful in predicting those patients who have the potential to have a long hospital stay. We feel that our findings are significant; however, further studies are needed to confirm the findings.
